# Gene alterations in monocytes are pathogenic factors for immunoglobulin a nephropathy by bioinformatics analysis of microarray data

**DOI:** 10.1186/s12882-018-0944-z

**Published:** 2018-07-20

**Authors:** Yingbo Guo, Wenfeng Gao, Danyang Wang, Weijing Liu, Zhongjie Liu

**Affiliations:** 1 Department of Nephropathy, Dongfang Hospital Affiliated to Beijing University of Chinese Medicine, Beijng, 100078 China; 2 Department of Urology, Dongzhimen Hospital Affiliated to Beijing University of Chinese Medicine, Beijng, 100700 China; 3 Department of Nephropathy and Endocrinology, Dongzhimen Hospital Affiliated to Beijing University of Chinese Medicine, No. 5 Haiyuncang, Dongcheng District, Beijng City, 100700 China

**Keywords:** Immunoglobulin a nephropathy, Network centrality analysis, Functional interaction, Toll-like receptor signaling, Apoptosis

## Abstract

**Background:**

Immunoglobulin A nephropathy (IgAN) is the most frequent primary glomerulopathy worldwide. The study aimed to provide potential molecular biomarkers for IgAN management.

**Methods:**

The public gene expression profiling GSE58539 was utilized, which contained 17 monocytes samples (8 monocytes samples isolated from IgAN patients and 9 monocytes samples isolated from healthy blood donors). Firstly, differentially expressed genes (DEGs) between the two kinds of samples were identified by limma package. Afterwards, pathway enrichment analysis was implemented. Thereafter, protein-protein interaction (PPI) network was constructed and key nodes in PPI network were predicted using four network centrality analyses. Ultimately, gene functional interaction (FI) was constructed according to expressions in each sample, and then module network was extracted from FI network.

**Results:**

A total of 678 DEGs were screened out, of these, 72 DEGs were identified as crucial nodes in PPI network that could well distinguish IgAN and healthy samples. In particular, *IL6*, *TNF*, *IL1B*, *PRKACA* and *CCL20* were closely related to pathways such as hematopoietic cell lineage, apoptosis and Toll-like receptor (TLR) signaling pathway. Moreover, 12 genes in the FI network belonged to the 72 identified key nodes, such as *CCL20*, *HDAC10*, *FPR2* and *PRKACA*, which were also key genes in 4 module networks.

**Conclusions:**

Several crucial genes were identified in monocytes of IgAN patients, such as *IL6*, *TNF*, *IL1B*, *CCL20*, *PRKACA*, *FPR2* and *HDAC10*. These genes might co-involve in pathways such as TLR and apoptosis signaling during IgAN progression.

## Background

Worldwide, immunoglobulin A nephropathy (IgAN) is the most frequent primary glomerulopathy. Reportedly, 20–50% of adults who suffered with IgAN would progress to end-stage renal diseases [1]. Therefore, it is pivotal for IgAN patients to identify predictors of prognosis. Numerous risk factors associated with IgAN progression have been reported. A study in Chinese population identifies three risk factors, including renal impairment, hypertension as well as advanced histological involvement [2]. Besides, another study reveals that expressions of renal leukocyte infiltrations and cytokines, such as leukocyte common antigen (LCA), CD3, CD68 and interleukin-1Beta (IL1B), are highly correlated with IgAN [3]. Currently, biochemical and genetic data indicate that aberrantly glycosylated IgA1 play significant roles in pathogenesis of IgAN [4–6]. Moreover, alteration on the glycan structure of IgA1 causes the deposition of nephritogenic immune complexes, which induce resident mesangial cells proliferation and extracellular matrix proteins expression, and subsequently lead to the loss of glomerular function [7]. Based on the pathogenesis, several biomarkers have been identified, such as levels of urinary secretory (sIgA) [8], serum galactose-deficient immunoglobulin A1 (Gd-IgA1) [9] and the tandem repeats polymorphism of *MUC20* gene [10]. However, cellular events involved in the IgAN pathogenesis are unclear.

Recently, it is found that abnormality of IgAN disease is related to IgA immune system and peripheral blood leucocytes, especially the peripheral blood mononuclear cells [11, 12]. Monocytes, a kind of the phagocytes that formed in bone marrow, can differentiate into macrophages and dendritic cells (DCs) in peripheral tissues. Monocytes have a crucial part in immune response and may contribute to the pathogenesis of IgAN [13]. Thus, a guideline for target therapy of IgAN will be obtained through identifying gene alterations in monocytes of IgAN patients. Moreover, Cox et al. uncover that the altered genes in IgAN monocytes are mainly associated with apoptotic pathway and mitochondrial dysfunction [13]. In particular, the expression of NADH: ubiquinone oxidoreductase core subunit S3 (NDUFS3) and TNF receptor superfamily member 1A (TNFRSF1A) proteins are upregulated, thus verifying the altered mitochondrial respiratory system and death receptor homeostasis. Additionally, the TNF expression in monocytes of IgAN patients are reduced compared with those in healthy blood donors (HBDs) [13]. However, other critical genes and their interaction have not been investigated.

In the present study, we re-analyzed GSE58539 profiling using a more comprehensive bioinformatics. After identifying the differentially expressed genes (DEGs) in monocytes between IgAN patients and HBDs, functional enrichment and protein-protein interactions (PPIs) network analyses were carried out, followed by key nodes prediction of the network through four network centrality analyses. Notably, in order to reveal potential interactions of DEGs that involved in similar functions and pathways, gene functional interaction (FI) network and the module network analyses were performed based on gene expressions of each sample. The study aimed to further uncover the pathogenesis and progression of IgAN, and thus provide potential molecular biomarkers for the diagnosis and targeting therapy of IgAN.

## Methods

### Data resource

The microarray data GSE58539 [13] was downloaded from Gene Expression Omnibus (GEO, http://www.ncbi.nlm.nih.gov/geo) database. This dataset contained 17 monocytes samples, including 8 monocytes samples isolated from IgAN patients (IgAN group) and 9 monocytes samples isolated from HBDs (healthy group). The platform of the dataset was Illumina HumanHT-12 V4.0 expression beadchip (Illumina, San Diego, California, USA).

### Data preprocessing

We used the robust multi-array average (RMA) method in Linear Models for Microarray Analysis (limma, http://www.bioconductor.org/packages/release/bioc/html/limma.html) package of R [14] to preprocess the non-normalized raw data by performing background correction, quantile normalization and microarray data condensation. Afterwards, the probe identification numbers (IDs) were transformed into gene symbols utilizing illuminaHumanv4.db [15] and annotate [16] software in R package and the probes were eliminated which did not correspond to gene symbols. Finally, the average value of different probes would serve as the final expression of the gene if different probes were mapped to the same gene.

### DEGs identification

Non-paired t-test method in limma package was utilized to calculate significance *p*-value of the DEGs between IgAN and healthy samples. The thresholds for DEG selection were *p*-value < 0.05 and log2|fold change| ≥ 0.58. Subsequently, coupled two-way clustering analysis (CTWC) was conducted using gplots tools [17] in R package.

### Enrichment analysis of the DEGs

The Database for Annotation, Visualization and Integration Discovery (DAVID, http://david.abcc.Ncifcrf.gov/) [18] tool was used to conduct Gene ontology (GO) and Kyoto Encyclopedia of Genes and Genomes (KEGG, http://www.genome.jp/kegg/pathway.html) [19] pathway enrichment analyses for DEGs. The number of enrichment genes (count number) ≥ 2 and *p*-value < 0.05 were chosen as cut-off criteria.

### Construction of the PPI network

The Search Tool for the Retrieval of Interacting Genes (STRING, http://string-db.org/) [20] database was used to predict potential interactions among proteins encoded by the DEGs. Relevant parameters were as follows: species was “Homo”, the input genes were DEGs and the PPI score (referred to medium confidence) was set as 0.4. A protein in the PPI network serves as a node. The network was visualized by the Cytoscape (http://cytoscape.org/) software [21].

### Prediction of key nodes in the PPI network

The topological property of PPI network makes it possible to investigate key genes in the network. Here, four network centrality analyses were performed to explore the key genes, including degree centrality, betweenness centrality, subgraph centrality and closeness centrality of key genes. Generally, degree was used for describing the importance of protein nodes in the network, and betweenness centrality is a kind of indicator that describes the global topological properties of the network. Besides, subgraph centrality was used to measure the importance of nodes in the network based on the combination of network topology and protein complex information. Closely centricity represented the closely connection degree of a certain node and all other nodes [22–25].

A cytoscape plug-in, CytoNCA [26], was used to perform the above analyses. Nodes with high values in the above four network centrality analyses were screened out to predict key genes, and the genes influence on sample clustering were observed using gplots packages. Detailed steps for the selection of key genes were: (1) the top ten genes with high values calculated by each network centrality analysis were selected and then were integrated; (2) if these integrated genes could not well distinguish the IgAN and healthy samples, more nodes were gradationally selected based on their ranked values to conduct the clustering analysis till they could distinguish completely the two kinds of samples. These key genes were then defined as feature genes of IgAN and healthy samples.

### FI network analysis

Based on gene expression value of each sample, the gene FI network was established using Cytoscape app-ReactomeFI [27]. The input dataset was the expression matrix of all DEGs. The FI network was analyzed utilizing ReactomeFI and the gene functional interaction in the PATHWAY of the Reactome database, thereafter modules from the FI network were obtained through Monte Carlo Localization clustering algorithm [28]. In addition, co-expression relationships of genes in each module were determined according to their expression value. The selection parameters in ReactomeFI network were module size ≥7 and average correlation ≥0.25. Subsequently, pathway enrichment analysis was carried out for each functional module to identify potential biological pathways associated with genes in each module, and the threshold for significant pathway selection was false discovery rate (FDR) < 0.05.

## Results

### DEGs identification

**Fig. 1:**
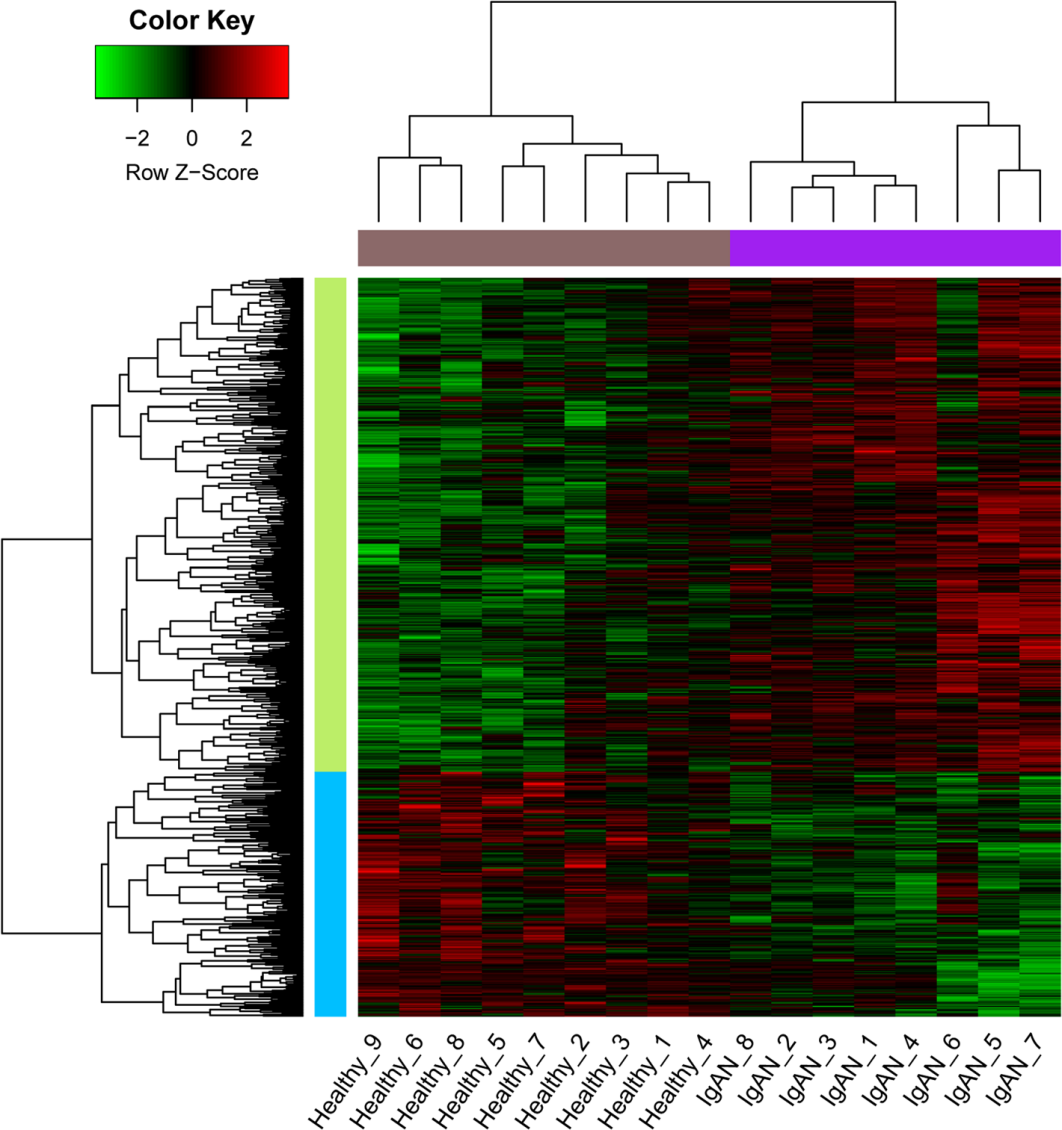
Heat map of clustering analysis of gene expressions in different samples. X-axis represents samples, and Y-axis represents gene expressions

Here, we obtained a total of 453 up-regulated and 225 down-regulated DEGs. As indicated in the clustering heat map (Fig. 1), these DEGs could well distinguish the IgAN and healthy samples completely.

### Pathway enrichment analysis of the DEGs

Unfortunately, the up-regulated DEGs were not enriched in any pathways. However, the down-regulated DEGs were significantly enriched in nine pathways. The enriched pathways were listed in Table 1, including hematopoietic cell lineage (pathway, *p*-value = 7.92 × 10^− 4^; which involved interleukin-6 (*IL6*), tumor necrosis factor (*TNF*) and interleukin 1 beta (*IL1B*)), NOD-like receptor signaling pathway (pathway, *p*-value = 8.22 × 10^− 3^; which involved *IL6*, *TNF* and *IL1B*), cytokine-cytokine receptor interaction (pathway, *p*-value = 1.91 × 10^− 2^; which involved *IL6*, *TNF*, *IL1B* and C-C motif chemokine ligand 20 (*CCL20*)), intestinal immune network for IgA production (pathway, *p*-value = 2.52 × 10^− 2^; which involved *IL6*) and apoptosis (pathway, *p*-value = 2.58 × 10^− 2^; which involved *TNF* and *IL1B*).

**Table 1:** Pathway enrichment analysis of the down-regulated differentially expressed genes in monocytes of IgAN patients

Term	Count	Genes	*P* value
hsa05322:Systemic lupus erythematosus	9	C1QA, HLA-DQB1, HIST1H2AC, TNF, HIST2H2BE, HIST1H2BH, H2AFY2, HIST3H2A, HLA-DQA2	3.52 × 10^−5^
hsa05332:Graft-versus-host disease	6	HLA-DQB1, IL6, TNF, IL1B, HLA-DQA2, IL1A	1.29 × 10^−4^
hsa04640:Hematopoietic cell lineage	7	CD55, IL6, TNF, IL1B, ITGB3, ITGA4, IL1A	7.92 × 10^−4^
hsa04940:Type I diabetes mellitus	5	HLA-DQB1, TNF, IL1B, HLA-DQA2, IL1A	2.00 × 10^−3^
hsa04621:NOD-like receptor signaling pathway	5	IL6, TNF, CXCL2, IL1B, TNFAIP3	8.22 × 10^−3^
hsa05020:Prion diseases	4	C1QA, IL6, IL1B, IL1A	1.02 × 10^− 2^
hsa04060:Cytokine-cytokine receptor interaction	9	IL6, TNF, CCL20, CXCL3, CXCL2, CSF2RB, IL1B, IL1A, TNFSF8	1.91 × 10^−2^
hsa04672:Intestinal immune network for IgA production	4	HLA-DQB1, IL6, ITGA4, HLA-DQA2	2.52 × 10^−2^
hsa04210:Apoptosis	5	TNF, CYCS, CSF2RB, IL1B, IL1A	2.58 × 10^−2^

### PPI network of the DEGs

**Fig. 2:**
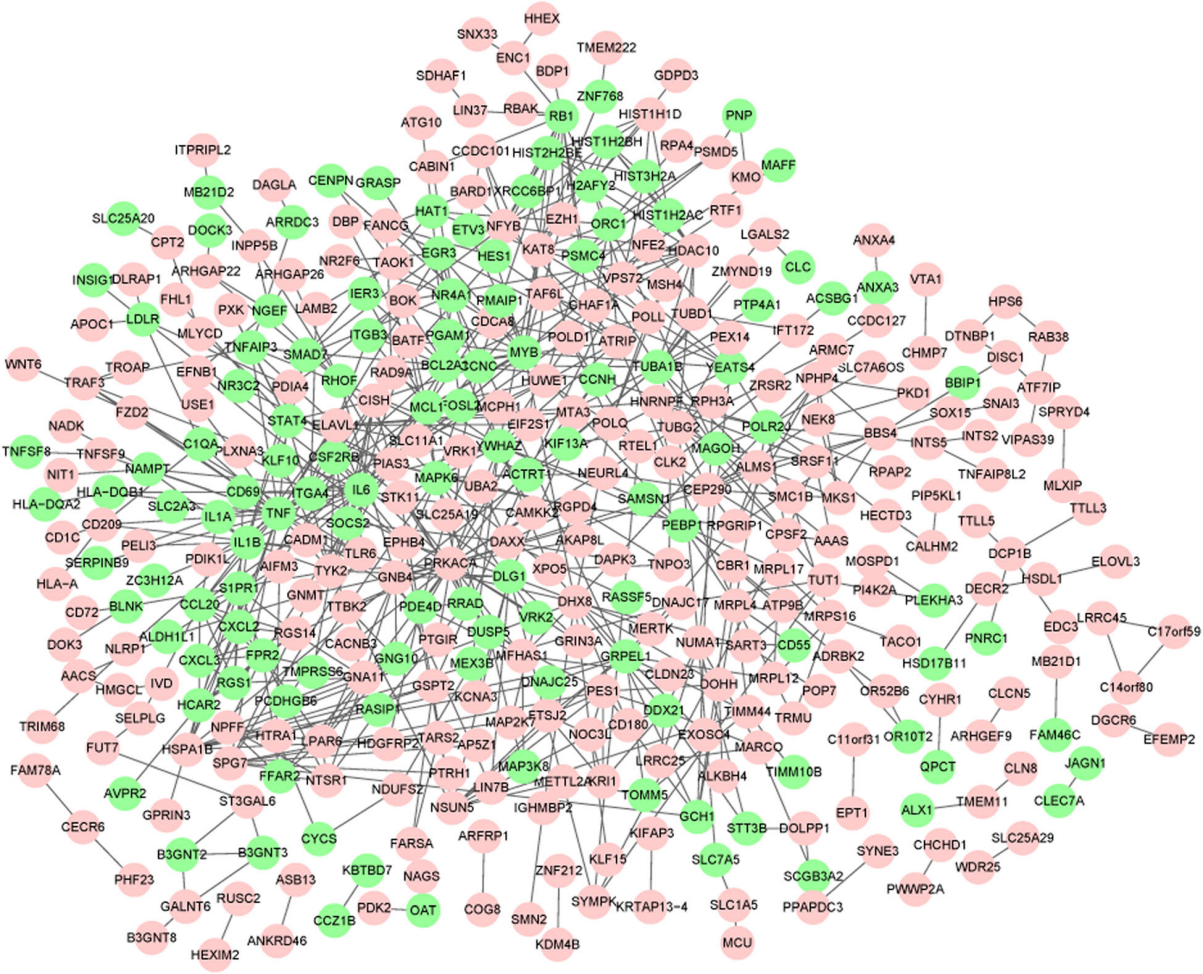
Protein-protein interaction network of the differentially expressed genes. Circle in red denotes upregulated genes, and in green denotes downregulated genes

As presented in Fig. 2, the PPI network with 379 nodes and 692 interactions was constructed. The hub nodes (whose degree > 10) mainly included TNF (degree = 31), PRKACA (degree = 26), IL6 (degree = 23), YWHAZ (degree = 19), MYB (degree = 15), TYK2 (degree = 14), FPR2 (degree = 13), IL1B (degree = 13), CCL20 (degree = 12), GNA11 (degree = 11).

### Key nodes in the PPI network

**Fig. 3:**
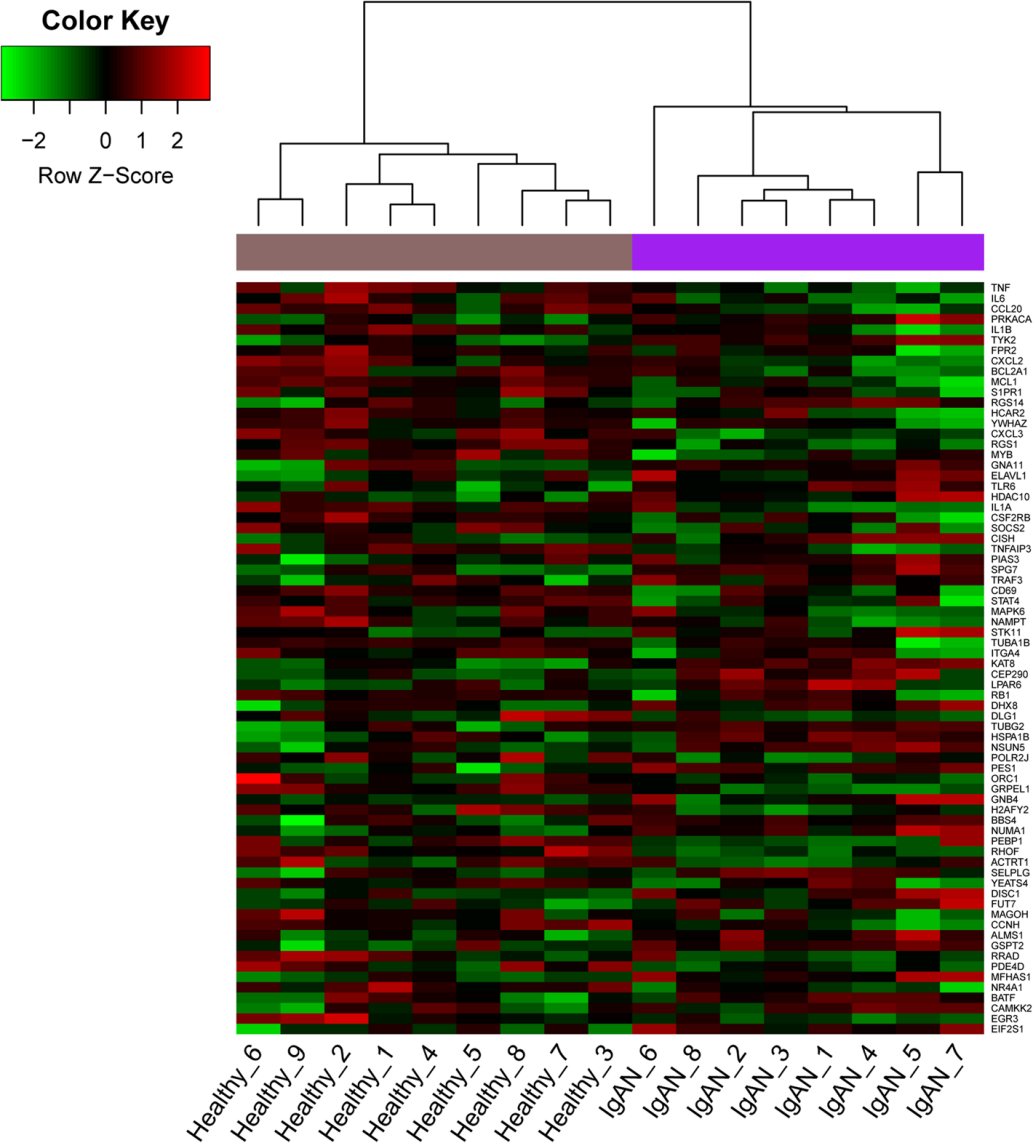
Heat map of clustering analysis of key genes predicted by four network centrality analyses in different samples. X-axis represents samples, and Y-axis represents gene expressions

Combined with the integrating results by four network centrality analyses, nodes with higher degree were used to cluster the two different kinds of samples. As a result, a total of 72 genes were identified that could well distinguish IgAN and healthy samples (Fig. 3). Among them, genes such as *IL6*, *TNF*, *IL1B*, *PRKACA*, *TYK2* and *CCL20* were closely related to five the pathways, including NOD-like receptor signaling pathway, cytokine-cytokine receptor interaction, hematopoietic cell lineage, apoptosis, and Toll-like receptor signaling pathway (Table 2).

**Table 2:** Enrichment analysis of hub genes in the protein-protein interaction network

Term	Genes	*P* value
hsa05020:Prion diseases	IL6, IL1B, PRKACA, HSPA1B, IL1A	1.30 × 10^−4^
hsa04630:Jak-STAT signaling pathway	TYK2, STAT4, IL6, SOCS2, PIAS3, CSF2RB, CISH	1.03 × 10^−3^
hsa04621:NOD-like receptor signaling pathway	IL6, TNF, CXCL2, IL1B, TNFAIP3	1.20 × 10^−3^
hsa05332:Graft-versus-host disease	IL6, TNF, IL1B, IL1A	3.15 × 10^−3^
hsa04060:Cytokine-cytokine receptor interaction	IL6, TNF, CCL20, CXCL3, CXCL2, CSF2RB, IL1B, IL1A	3.31 × 10^−3^
hsa04640:Hematopoietic cell lineage	IL6, TNF, IL1B, ITGA4, IL1A	4.00 × 10^−3^
hsa04210:Apoptosis	TNF, CSF2RB, IL1B, PRKACA, IL1A	4.17 × 10^−3^
hsa04620:Toll-like receptor signaling pathway	IL6, TNF, IL1B, TLR6, TRAF3	7.08 × 10^−3^
hsa04940:Type I diabetes mellitus	TNF, IL1B, IL1A	4.07 × 10^−2^

### FI network analysis

**Fig. 4:**
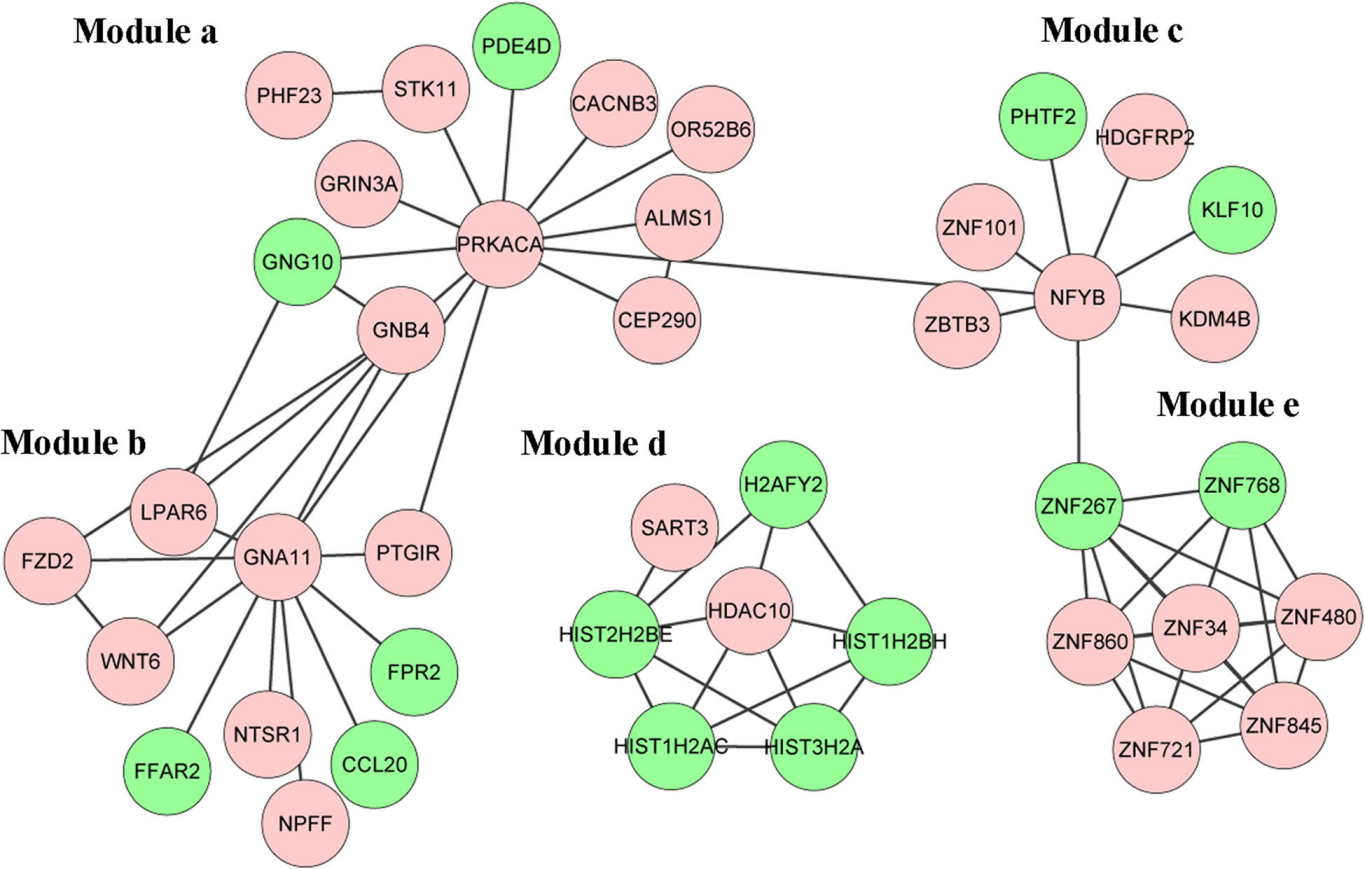
Gene functional interaction network of the differentially expressed genes. Circle in red denotes upregulated genes, and in green denotes downregulated genes

The FI network of the DEGs was constructed utilizing ReactomeFI, which included 42 genes and 71 interaction edges (Fig. 4). Moreover, five modules (module a-e) were extracted from the FI network, and the absolute average correlation of genes in module a-e was 0.58, 0.4222, 0.5069, 0.4709 and 0.4275, respectively. Genes such as *PRKACA* in module a was enriched in integration of energy metabolism, morphine addiction and glutamatergic synapse pathways; in module b, genes such as *CCL20*, *FPR2* and *GNA11* were related to GPCR ligand binding, GPCR downstream signaling and Gastrin-CREB signaling pathway via PKC and MAPK pathways; in module c, the gene *NFYB* was highly associated with RNA binding-related pathways; while in module d, the gene *HDAC10* was significantly enriched in two pathways, alcoholism and chromatin modifying enzymes (Table 3) . Genes in module e were not enriched in any pathways.

**Table 3:** Enrichment analysis of genes in each functional interaction network module

Module	GeneSet	FDR	Genes
a	Integration of energy metabolism(R)	8.62 × 10^−5^	STK11, GNG10, GNB4, PRKACA
a	Morphine addiction(K)	8.62 × 10^− 5^	GNG10, GNB4, PDE4D, PRKACA
a	Glutamatergic synapse(K)	1.39 × 10^−4^	GNG10, GNB4, PRKACA, GRIN3A
b	GPCR ligand binding(R)	7.31 × 10^−7^	PTGIR, CCL20, LPAR6, FFAR2, FPR2, NTSR1, NPFF
b	GPCR downstream signaling(R)	4.07 × 10^−6^	PTGIR, CCL20, LPAR6, FFAR2, GNA11, FPR2, NTSR1, NPFF
b	Gastrin-CREB signaling pathway via PKC and MAPK(R)	6.99 × 10^−6^	LPAR6, FFAR2, GNA11, FPR2, NTSR1, NPFF
c	overview of telomerase rna component gene hterc transcriptional regulation(B)	1.29 × 10^−2^	NFYB
c	DNA Double Strand Break Response(R)	1.62 × 10^−2^	KDM4B
c	Regulation of cholesterol biosynthesis by SREBP (SREBF)(R)	1.62 × 10^−2^	NFYB
d	Alcoholism(K)	1.74 × 10^−10^	HIST1H2AC, HIST2H2BE, H2AFY2, HIST1H2BH, HDAC10, HIST3H2A
d	Systemic lupus erythematosus(K)	6.92 × 10^−9^	HIST1H2AC, HIST2H2BE, H2AFY2, HIST1H2BH, HIST3H2A
d	Chromatin modifying enzymes(R)	1.55 × 10^−4^	HIST2H2BE, HIST1H2BH, HDAC10

*FDR* false discovery rate, *R* data from Reactome database, *K* data from KEGG database, *B* data from BioCarta database

Notably, we found that 12 genes in the FI network also belonged to the hub genes, such as *CCL20*, *FPR2*, and *PRKACA*.

## Discussion

In the present study, a total of 72 crucial nodes in the PPI network were identified via re-analyzing the dataset GSE58539, which could well distinguish the IgAN and healthy samples. Among which, genes such as *IL6*, *TNF*, *IL1B*, *PRKACA*, and *CCL20* were closely related to the following pathways: NOD-like receptor signaling pathway, cytokine-cytokine receptor interaction, hematopoietic cell lineage, apoptosis and Toll-like receptor signaling pathway. Moreover, 12 genes in the FI network belonged to the 72 identified key nodes, such as *CCL20*, *HDAC10*, *FPR2* and *PRKACA*. Besides, the 12 genes were also the key genes in 4 module networks correlating with pathways of integration of energy metabolism (module a), GPCR-related pathways (module b), RNA binding-related pathways (module c), alcoholism and chromatin modifying enzymes (module d).

The cytokine encoded by *IL6* has great roles in inflammation and regulation of immune response [29]. Toll-like receptors (TLRs) are major factors that initiate the immune reaction. Most TLRs promote immune response (including innate and adaptive) via inducing expression of proinflammatory cytokines [30]. Increased TLRs, such as TLR-4, has been detected in circulating monocytes of patients with IgAN [31]. Expression of IL6 protein is also increased in mouse proximal tubular epithelial cells, accompanying by the upregulation of *TLR4* mRNA [32]. IL1B, encoded by *IL1B* gene, is a member of interleukin 1 cytokine family and crucial for the regulation of inflammatory response [33]. In response to the external infections, gene expressions of the proinflammatory cytokines (e. g. *IL1A*, *IL1B* and *IL6*) are always upregulated simultaneously [34, 35]. In particular, *IL1B* is implicated in the *TLR-4* induced immune response in chronic pain [36]. In our study, *IL6* and *IL1B* were both downregulated and enriched in TLR signaling pathway. These results suggested that *IL6* and *IL1B* might be co-regulated in TLR signaling pathway and contribute to the abnormality of the immune response in monocytes of IgAN patients.

TNF is a multifunctional proinflammatory cytokine. Reportedly, TNF expression is dramatically increased in *Mycoplasma penetrans*-infected IgAN mice model, and the protein is proposed to involve in the induction of renal damage in IgAN [37]. Moreover, levels of serum TNF receptors are also elevated in IgAN patients compared with healthy control [38]. However, Cox et al. uncover that *TNF* expression is obviously reduced in monocytes of IgAN patients, compared with those of HBDs [13]. The finding indicates the downregulated TNF may lead to the monocytes apoptosis. Moreover, the inhibition of TNF-α is proposed as a causative factor of IgAN [39]. Therefore, it might be speculated that the apoptosis of monocytes induced by downregulation of TNF contribute to IgAN progression.

CCL20 is a small cytokine that also involves in immune regulation and inflammation [40]. Combination of CCL20 with CCR6 (the CCL20 receptor) cause the recruitment of leukocyte subsets, which finally promote immune-mediated kidney damage [41]. Additionally, *CCL20* is one of the chemokines that take part in the host response to pathogens invasions by activating inflammatory cells, and it has the similar effects on monocytes [42]. Therefore, the downregulated *CCL20* in monocytes of IgAN patients might cause alteration in immune response, and thereby influence the IgAN development.

Three novel genes, protein kinase, CAMP-dependent, catalytic, alpha (*PRKACA*), formyl peptide receptor 2 (*FPR2*) and histone deacetylase 10 (*HDAC10*) were firstly predicted in monocytes of IgAN. PRKACA protein encoded by *PRKACA* gene is one subunit of protein kinase A that participates in apoptosis. At present, *PRKACA* amplification is served as a method for identifying genetic defect correlated with Cushing’s syndrome [43]. Somatic mutations of *PRKACA* have been detected in adenomas of the adrenal cortex [44]. Moreover, *PRKACA* mediates apoptosis-related signaling pathways in many cancer diseases, such as breast cancer and follicular thyroid cancer cells [45, 46]. In the present study, *PRKACA* was up-regulated and significantly enriched in apoptosis pathway, suggesting it might exert its function in monocytes via regulating apoptosis during IgAN progression. *FPR2* is known to activate the G-protein coupled receptor and N-formyl peptide receptor. *FPR*2 is found in adipose tissues as a receptor for the pro-resolving mediators [47], which contribute to the restoration of in adipose inflammation and treatment of obesity-related glomerulopathy [48]. *HDAC10*, containing two catalytic sites, is highly expressed in numerous human tissues such as kidney [49]. In lung cancer, decreased *HDAC10* is associated with the advanced stage and adverse outcome [50]. However, there are rare reports on the relationship of IgAN and *HDAC10*. In the current study, *FPR2* and *HDAC10* were hub up-regulated genes in both PPI network and FI network, implying they might co-function in monocytes of IgAN patients. One limitation of this study is the lack of expression validation. However, we will do more experiments to verify our conclusions once we collect the samples in the future.

## Conclusions

In conclusion, several crucial genes were identified in monocytes of IgAN patients, such as *IL6*, *TNF*, *IL1B*, *CCL20*, *PRKACA*, *FPR2* and *HDAC10*. They might have co-functions and their dysregulations might alter activities of pathways such as TLR and apoptosis signaling, which might finally promote IgAN progression. The study is of great value for the prediction of key regulators in monocytes of IgAN and the identification of targeting therapeutic management for IgAN.

## Abbreviations

DCs: Dendritic cells
DEGs: Differentially expressed genes
FDR: False discovery rate
FI: Functional interaction
*FPR2*: Formyl peptide receptor 2
Gd-IgA1: Galactose-deficient immunoglobulin A1
GEO: Gene Expression Omnibus
HBDs: Healthy blood donors
*HDAC10*: Histone deacetylase 10
IDs: Identification numbers
IgAN: Immunoglobulin A nephropathy
IL1B: interleukin-1Beta
*IL6*: Interleukin-6
KEGG: Kyoto encyclopedia of genes and genomes
LCA: Leukocyte common antigen
PPI: Protein-protein interaction
RMA: Robust multi-array average
STRING: Search tool for the retrieval of interacting genes
TLRs: Toll-like receptors
TNF: Protein Kinase, CAMP-Dependent, Catalytic, Alpha
TNF: Tumor necrosis factor
